# Supplementary material for: SCCmec transformation requires living donor cells in mixed biofilms
Source: Biofilm. 2024 Feb 16;7:100184. doi: 10.1016/j.bioflm.2024.100184 (PMC10909703; doi:10.1016/j.bioflm.2024.100184)
Supplement: Multimedia component 1 [file mmc1.pdf]

Fig S1

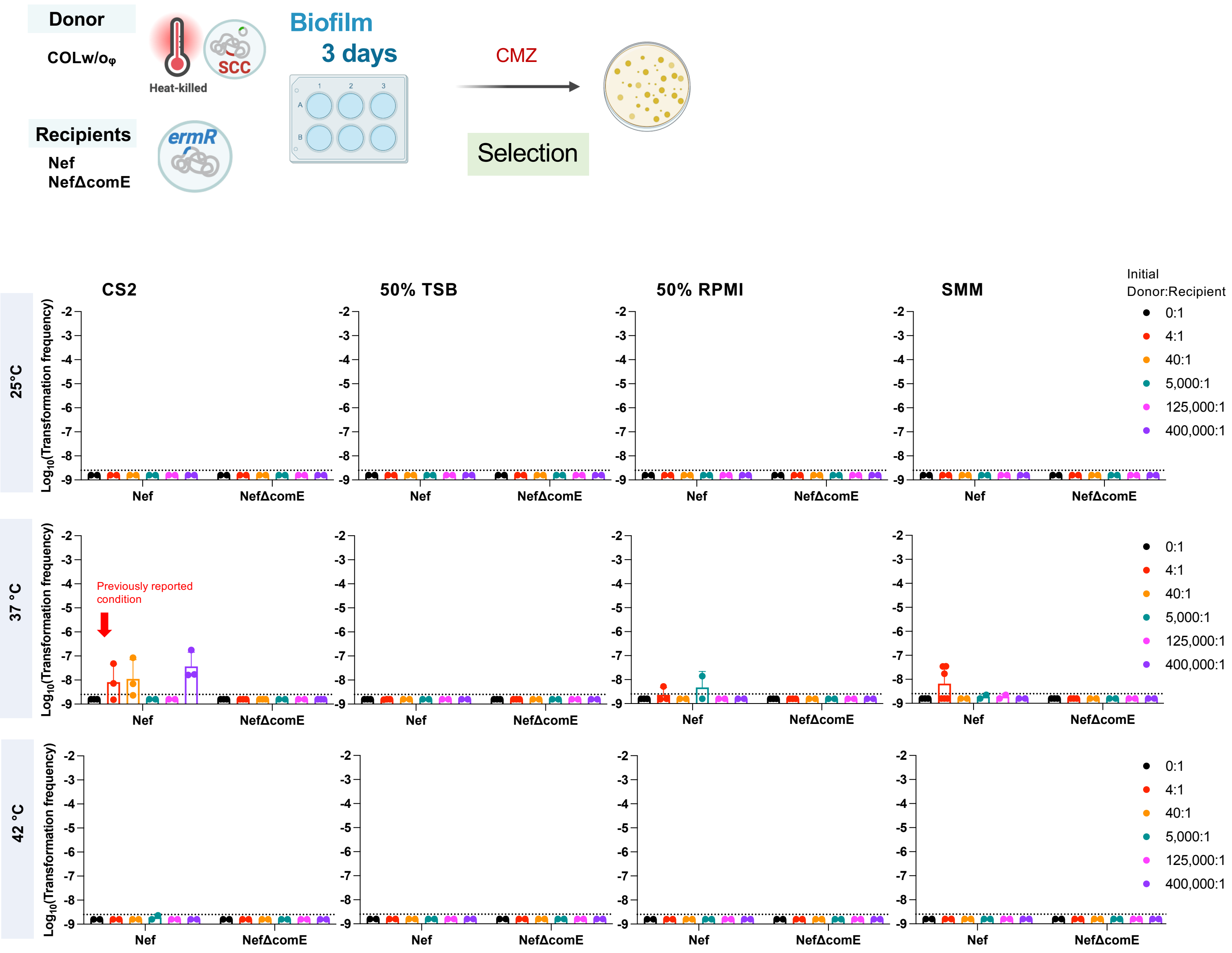

Figure S1. Transformation efficiency of SCC*mec* in the biofilms in different conditions using heat-killed donor.

The recipient cells (Nef or NefΔcomE) were statically grown in different media at different temperatures for 3 days. An initial fixed amount of the heat-killed COLw/o$_{\varphi}$ donor cells was used, generating the following donor-to-recipient ratios: 0:1, 3:1, 40:1, 5000:1, 25000:1, and 125000:1. Transformation efficiencies were determined after 3 days by cefmetazole (CMZ) selection.

The mean of at least n = 2 independent experiments is shown with SD. Data points represent independent experiments. The dotted lines represent the detection limit.

Fig S2

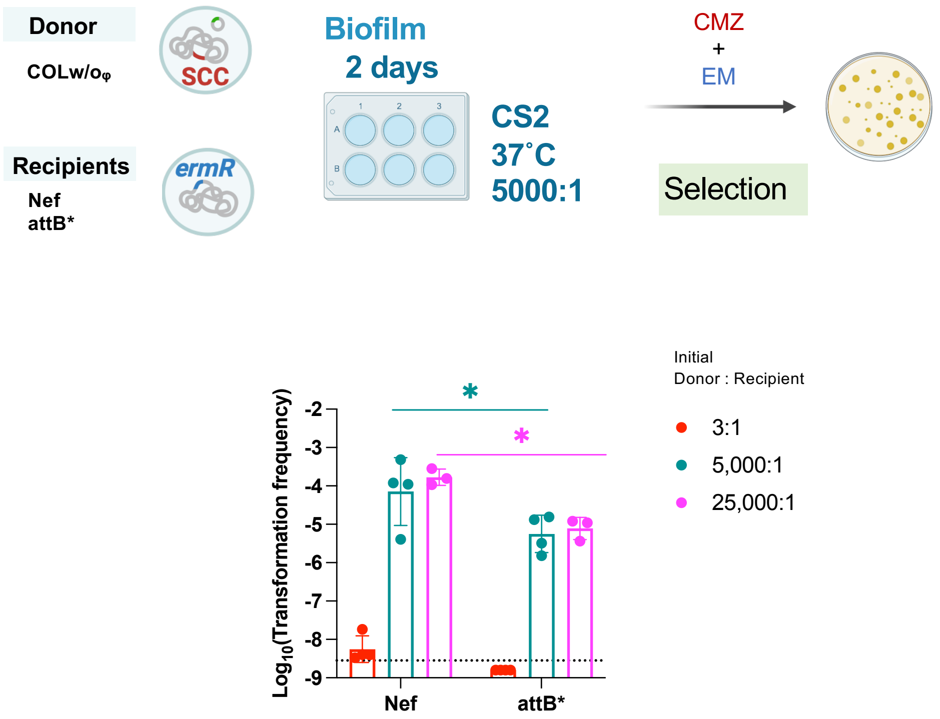

Figure S2. The *attB site* is important for SCC*mec* transformation.
Living donor COLw/o$_{\varphi}$ cells were added to recipient cells (Nef or attB*) in a 3:1, 5000:1, or 25000:1 ratio. The cells were statically grown in CS2 for 2 days at 37°C. Transformants were selected by erythromycin (EM) and cefmetazole (CMZ) The mean of at least n = 3 independent experiments is shown with SD. Data points represent independent experiments. The dotted lines represent the detection limit. The same data set at 5 000:1 ratio was used in Fig. 3A. Statistical significance was determined by 2-way ANOVA followed by Tuckey's multiple comparison tests. *P<0.05.

Fig S3

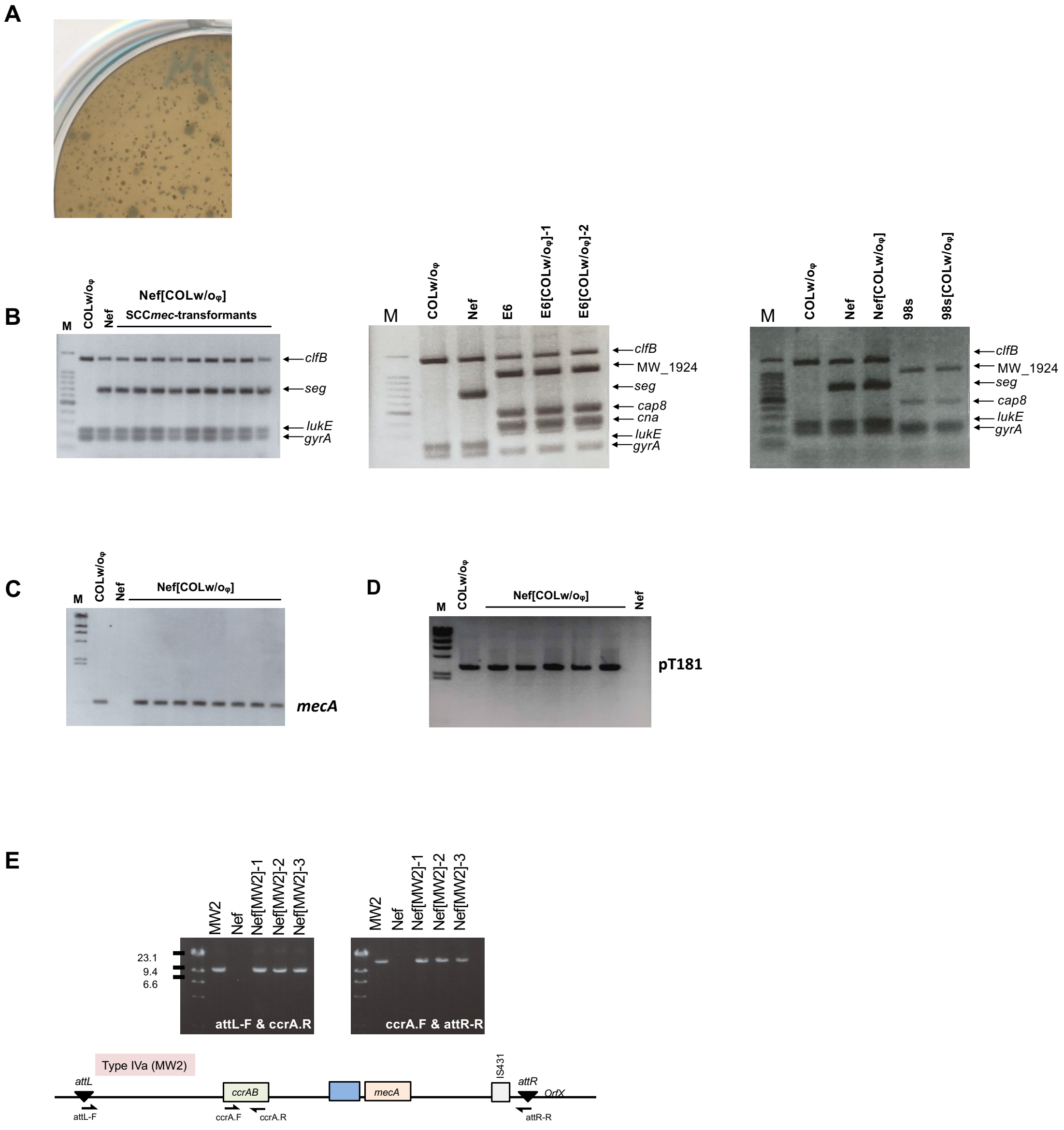

Figure S3. Confirmation of transformants by PCR.

(A) Representative photo of SCC*mec*-transformants using COLw/o$_{\varphi}$ as donor and Nef as the recipient in a 5000:1 ratio, from 2-day biofilm.

(B) Transformants share the same genetic background as the recipient, as validated by multiplex PCR: The amplification patterns are the same between transformants and each original recipient. M: 100 bp DNA ladder (Takara).

(C) The SCC*mec*-transformants have the *mecA* gene. Genomic DNA of the donor and recipient was used as positive and negative controls, respectively.

(D) Transformants carry the pT181 plasmid. Genomic DNA of the donor and recipient was used as positive and negative controls, respectively. M: λ-*Hin*dIII DNA ladder (Takara).

(E) SCC*mec* IVa amplification in the Nef transformants obtained from 2-day biofilm using a 5000:1 donor-to-recipient ratio. The schematic structure of the SCC and primer locations indicated by arrows are shown. DNA of COLw/o$_{\varphi}$ donor and Nef recipient was used for positive and negative controls. Suffixes (1), (2), and (3) represent transformants obtained from three independent experiments. M: DNA marker, λ-*Hin*dIII.

Fig S4

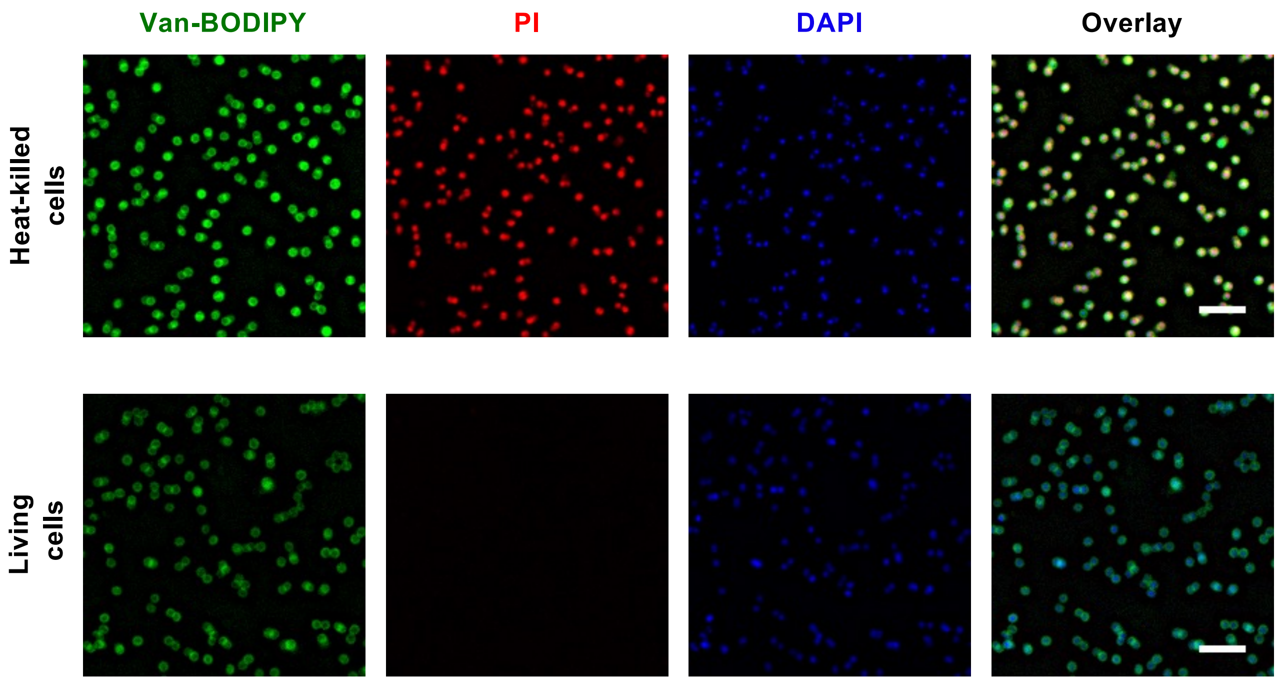

Figure S4. DNA is retained within the cell wall in heat-killed donor cells.
Cell walls were stained by Van-BODIPY. Dead cells were visuslized by propidium iodide (PI), and the cellular nucleoids were visualized by DAPI. Scale bars = 5 μm.

Fig S5

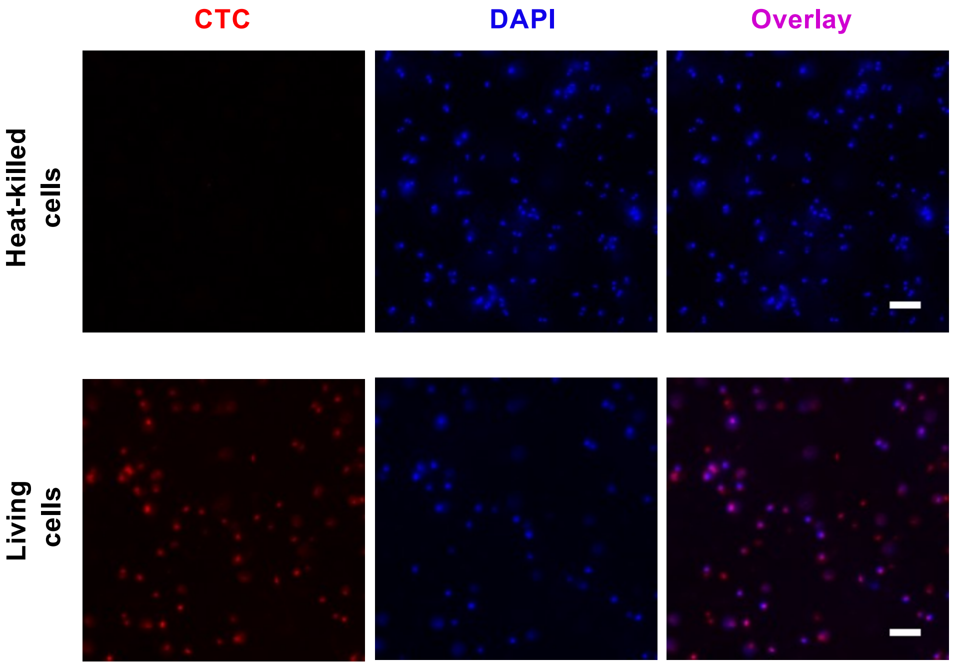

Figure S5. CTC and DAPI double staining of *S. aureus* donor cells.
Cells capable of respiration were stained by CTC. Cellular nucleoids were visualized by DAPI. Scale bars = 5 µm.

Fig S6

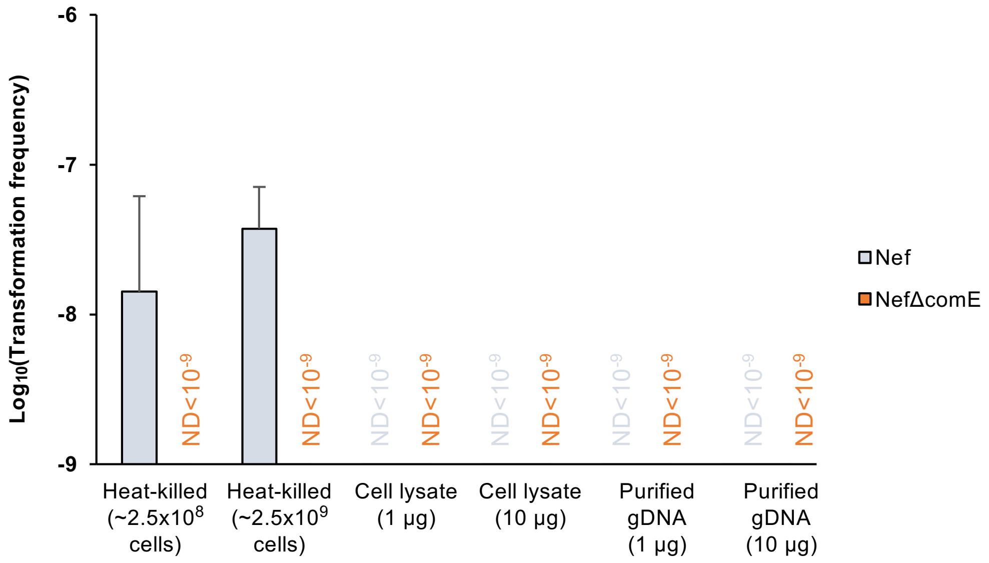

Figure S6. Transformation efficiency of SCC*mec* in the biofilm using heat-killed cells, cell lysate, and purified DNA as donors.

The recipient cells Nef or NefΔcomE were statically grown in CS2 for 3 days. Heat-killed COLw/o$_{\varphi}$ donor cells tested were ~2.5 × 10^8 or ~2.5 × 10^9 CFU equivalent. The quantity of genome DNA is estimated to be ~1 μg in ~2.5x10^8 cells. The cell lysate containing 1 μg or 10 μg DNA or purified genomic DNA (gDNA) of 1 μg or 10 μg were also used as donors. Transformation efficiencies were determined after 3 days by selection with cefmetazole. The mean of n = 2 independent experiments is shown with SD. ND, none detected (detection limit is 10^{-9}).

Method

To prepare the cell lysate and purified DNA for the transformation assays (Fig. S6), overnight cultures of COLw/oφ donor were diluted 20-fold in TSB and grown for 3 h at 37˚C with shaking. Cells were washed, harvested in PBS (~10^9 CFU/mL), and lysed by beads using the Desktop Bead Crusher Shakeman 6 (4350 rpm, 60 sec, 1 run). The genomic DNA was further purified from this cell lysate using the standard phenol-chloroform method. The DNA was quantified using Quanti-iT^{TM} Pico Green dsDNA Assay kit (Invitrogen). Cell lysate containing 1 or 10 μg DNA or the purified genomic DNA was added to 750 mL of log-phase recipient cells (~10^8 CFU/mL) in the 6-well plate, and the total volume of growth medium (CS2) was adjusted to 1.5 mL per well. The biofilms were statically incubated for 3 days at 37˚C, and the medium was refreshed every 24 h. The biofilms were collected by pipetting and poured into BHI agar supplemented with 4 μg/mL cefmetazole for the selection of SCC*mec*-transformants.

Table S1

Table S1. The number of transformants in biofilm obtained from living COLw/o$_{\varphi}$ donor cells (from Fig. 2).

	1-day biofilm		2-day biofilm		3-day biofilm	
Initial Donor:Recipient	**Nef**	**NefΔcomE**	**Nef**	**NefΔcomE**	**Nef**	**NefΔcomE**
**3:1**	**3** ± 1.4 (n=2), ND (n=2)	ND (n=4)	**2** ± 1.5 (n=3)	0 (n=3)	**2** ± 1.2 (n=3), ND (n=1)	ND (n=4)
**40:1**	**29** ± 19 (n=5)	ND (n=5)	**699** ± 1617 (n=6)	0 (n=6)	**131** ± 86 (n=5)	ND (n=5)
**5,000:1**	**26** ± 36 (n=5)	ND (n=5)	**5,234** ± 5,896 (n=5)	0 (n=4)	**1142** ± 924 (n=5)	ND (n=5)
**25,000:1**	**4** ± 1.4 (n=2), ND (n=2)	ND (n=5)	**9,569** ± 15,485 (n=5)	0 (n=4)	**514** ± 610 (n=4)	ND (n=4)
**125,000:1**	12 (n=1), ND (n=3)	ND (n=5)	**711** ± 820 (n=4)	0 (n=3)	**283** ± 260 (n=4)	ND (n=4)

The mean of n independent experiments is shown ± SD.

Table S2

Table S2. The number of transformants in a 2-day biofilm obtained from living COLw/o$_{\varphi}$Δcls1-cm^{R} donor cells.

	Initial Donor:Recipient	Transferred element	Nef	NefΔcomE	Transferred element	Nef	NefΔcomE	Transferred element	Nef	NefΔcomE
**Living donor cells**	**3:1**	**SCC**	1 (n=1), ND (n=2)	ND (n=3)	**pT181 plasmid**	ND (n=3)	ND (n=3)	**Chromosomal cmR**	ND (n=3)	ND (n=3)
	**5,000:1**		**596** ± 585 (n=3)	ND (n=3)		**208** ± 85 (n=3)	ND (n=3)		**10,835** ± 3,071 (n=3)	ND (n=3)
	**125,000:1**		**16** ± 13 (n=3)	ND (n=3)		**43** ± 53 (n=3)	ND (n=3)		**11,881** ± 3,237 (n=3)	ND (n=3)
**Heat-killed donor cells**	**3:1**	**SCC**	ND (n=2)	ND (n=2)	**pT181 plasmid**	ND (n=2)	ND (n=2)	**Chromosomal cmR**	ND (n=2)	ND (n=2)
	**5,000:1**		**3** (n=1), ND (n=1)	ND (n=2)		ND (n=2)	ND (n=2)		ND (n=2)	ND (n=2)
	**125,000:1**		ND (n=2)	ND (n=2)		ND (n=2)	ND (n=2)		ND (n=2)	ND (n=2)

The mean of n independent experiments is shown ± SD.

Table S3. Baterial strains and plasmids used in this study

Strains	Description	Source
RN4220	Derivative of 8325-4, restriction minus, modification plus	Kreiswirth et al, 1983
Nef	N315ex w/oφ (N315ex cured of the φN315 prophage), 4 erm^R genes in genome, clonal complex 5	Morikawa et al, 2012
attB*	Nef with mutated *attB* site	Maree et al., 2022
NefΔcomE	Nef lacking *comE* operon	Morikawa et al, 2012
COLw/oφ	COL strain cured of the φL54a prophage, clonal complex 8, SCC*mec* I	Morikawa et al, 2012
COLw/oφΔcls1-cm^R	COLw/oφ, lacking *cls1* gene, cm^R	This study
COLw/oφ-Δccr	COLw/oφ, lacking *ccr* genes	This study
98s	MSSA clinical isolate, erm^R, clonal complex unable to categorize by multiplex PCR	This study
E1	MSSA clinical isolate, erm^R, clonal complex 30	This study
E2	MSSA clinical isolate, erm^R, clonal complex 8	This study
E3	MSSA clinical isolate, erm^R, clonal complex 8	This study
E4	MSSA clinical isolate, erm^R, clonal complex 1	This study
E5	MSSA clinical isolate, erm^R, clonal complex 1	This study
E6	MSSA clinical isolate, erm^R, clonal complex unable to categorize by multiplex PCR	This study
E7	MSSA clinical isolate, erm^R, clonal complex 1	This study
E8	MSSA clinical isolate, erm^R, clonal complex 133	This study
9s	MSSA clinical isolate, clonal complex unable to categorize by multiplex PCR	Maree et al., 2022
9sΔcomE	9s mutant lacking c*omE* operon	Maree et al., 2022
9s-ermR	MSSA clinical isolate, erm^R by transduction from Nef	This study
9sΔcomE-ermR	9s lacking *comE* operon, erm^R by transduction from Nef	This study
MR-CoNS4	Methicillin resistant *S. capitis*, SCC*mec* nontypeable	This study
MR-CoNS11	Methicillin resistant *S. epidermidis*, SCC*mec* IVa / I	Maree et al., 2022
MR-CoNS18	Methicillin resistant *S. capitis*, SCC*mec* I	This study
MW2	Methicillin resistant *S. aureus*, SCC*mec* IVa	CDC 1999
DH5α	*E. coli* plasmid cloning host	Toyobo Ltd., Japan

Plasmids

pMADcat	pMAD derivative, Amp^R (*E. coli*), Erm^R, Cm^R (*S. aureus*)	Tsai et al, 2011
pMADcat1155	Vector for deletion of *cls1* locus, Amp^R (*E. coli*), Erm^R, Cm^R (*S. aureus*)	Tsai et al, 2011
pMADcat-Δccr	Vector for deletion of *ccrAB1* locus, Amp^R (*E. coli*), Erm^R, Cm^R (*S. aureus*)	This study

References

Kreiswirth, B. N. et al. The toxic shock syndrome exotoxin structural gene is not detectably transmitted by a prophage. Nature 305, 709–712 (1983).

Maree, M. et al. Natural transformation allows transfer of SCCmec-mediated methicillin resistance in Staphylococcus aureus biofilms. Nat Commun 13, 2477, doi: 10.1038/s41467-022-29877-2 (2022).

Morikawa, K. et al. Expression of a cryptic secondary sigma factor gene unveils natural competence for DNA transformation in *Staphylococcus aureus*. PLoS Pathog 8, e1003003, doi:10.1371/journal.ppat.1003003 (2012).

Tsai, M. et al. *Staphylococcus aureus* requires cardiolipin for survival under conditions of high salinity. BMC Microbiol 11, 13, doi:10.1186/1471-2180-11-13 (2011).

CDC. 1999. Four pediatric deaths from community-acquired methicillin- resistant Staphylococcus aureus—Minnesota and North Dakota, 1997–1999. MMWR Morb Mortal Wkly Rep 48:707–710.

Table S4. List of primers used in this study

Primer	Sequence	Source
**Δccr mutant construction**		
ccr-A	GCGGGATCCAAAAGCTGTGGCACTAAACCA	This study
ccr-B	TTGCTTTGTAGTGCTCTGTCG	This study
ccr-C	CGACAGAGCACTACAAAGCAACGCTAACAAATCATGGCTCA	This study
ccr-D	GCCGTCGACCATCTTCTTTCAGTCTAAAAATGTGT	This study
**Δccr mutant check**		
ccr-E	GCCCAAGACAAAATTGATGC	This study
ccr-F	CCATGACCTTTGGCATAAGA	This study
**SCC*mec* amplification**		
mecAF	GTAGTTGTCGGGTTTGGT	Morikawa et al, 2012
mecAR	GGTATCATCTTGTACCCA	Morikawa et al, 2012
Xsau325	GGATCAAACGGCCTGCACA	Morikawa et al, 2012
3.0-R	CTCAGACAGCAATTTCCCG	Morikawa et al, 2012
attL-F	ACTTATGATACGCCTCTGCTT	Maree et al, 2022
attR-R	AGAAGCTTATCATAAGTAATGAGG	Maree et al, 2022
ccrA.F	TGAATGCTTCACGCTTTGTC	Maree et al, 2022
ccrA.R	TTGCGTTTGTTCTCTGAACG	Maree et al, 2022
plsR	TACTCAAGGGAATGGCCAAG	Maree et al, 2022
plsF	TCACACCATCTGCACCATTT	Maree et al, 2022
ccrA1R	GATTGCGATGAAGTCGGTTT	Maree et al, 2022
ccrA1F	ATCGAGGCATTAGCCAAAAA	Maree et al, 2022
**CC typing by multiplex PCR**		
clfBF	AACAGAGCCAGCTTCAACAAATGA	Schwalm et al, 2011
clfBR	GTCTTTCGGATTTACTGCTGAATC	Schwalm et al, 2011
MW_1924F	TCACGAAGTCGAACGAAGAA	Schwalm et al, 2011
MW_1924R	GCTGAACGCTCTTCTGCTTC	Schwalm et al, 2011
segF	AGAATCAACAACTTTATTATCTCCG	Schwalm et al, 2011
segR	TATGTGAATGCTCAACCCGA	Schwalm et al, 2011
arcDF	TGCCATTGATGGATTAGCAA	Schwalm et al, 2011
arcDR	GTTTTTCAAGTGCTTGGGGA	Schwalm et al, 2011
cap8F	GGAGGAAATGACGATGAGGA	Schwalm et al, 2011
cap8R	TGTCACCCTGCTAGCATCAA	Schwalm et al, 2011
cnaF	TGCTGTCCACCTTGAATCTG	Schwalm et al, 2011
cnaR	GTTATTACGCCAGACGGAGC	Schwalm et al, 2011
lukEF	GCATTATGCTTTCTTTCTGCG	Schwalm et al, 2011
lukER	AATGGTCCAACAGGTTCAGC	Schwalm et al, 2011
gyrAF	AAGGTGTTCGCTTAATTCGC	Yoshida et al, 2011
gyrAR	ATTGCATTTCCTGGTGTTTC	Yoshida et al, 2011
**Transformants check**		
pt181-F	GGTCCTTCAATAGGGGGAAT	This study
pt181-R	CACAGTATGTGCGTCCAAC	This study
cmR-F	TCCTGCATGATAACCATCACA	This study
cmR-R	AATGGTTCGGGGAAATTGTT	This study

Underlined, restriction site included in the primer.

References

Maree, M. et al. Natural transformation allows transfer of SCCmec-mediated methicillin resistance in *Staphylococcus aureus* biofilms. Nat Commun 13, 2477, doi: 10.1038/s41467-022-29877-2 (2022).

Morikawa, K. et al. Expression of a cryptic secondary sigma factor gene unveils natural competence for DNA transformation in *Staphylococcus aureus*. PLoS Pathog 8, e1003003, doi:10.1371/journal.ppat.1003003

Schwalm, N. D., Verghese, B. & Knabel, S. J. A novel multiplex PCR method for detecting the major clonal complexes of MRSA in nasal isolates from a Pennsylvania hospital. Journal of microbiological methods 86, 379-382, doi:10.1016/j.mimet.2011.05.024 (2011).

Yoshida, Y. et al. Bacitracin sensing and resistance in *Staphylococcus aureus*. FEMS Microbiology Letters 320, 33–39 (2011).
